# Recommendations for radioembolisation after liver surgery using yttrium-90 resin microspheres based on a survey of an international expert panel

**DOI:** 10.1007/s00330-017-4889-6

**Published:** 2017-07-03

**Authors:** Morsal Samim, Linde M. van Veenendaal, Manon N. G. J. A. Braat, Andor F. van den Hoven, Richard Van Hillegersberg, Bruno Sangro, Yung Hsiang Kao, Dave Liu, John D. Louie, Daniel Y. Sze, Steven C. Rose, Daniel B. Brown, Hojjat Ahmadzadehfar, Edward Kim, Maurice A. A. J. van den Bosch, Marnix G. E. H. Lam

**Affiliations:** 1 Department of Surgery, University Medical Center Utrecht, Utrecht, The Netherlands; 2 Department of Radiology and Nuclear Medicine, University Medical Center Utrecht, Utrecht, The Netherlands; 3 Liver Unit, Clinica Universidad de Navarra-IDISNA and CIBEREHD, Pamplona, Spain; 4 Department of Nuclear Medicine, Cabrini Hospital, Melbourne, Australia; 5 Department of Radiology, Vancouver General Hospital. University of British Columbia, Vancouver, British Columbia Canada; 6 Division of Interventional Radiology, Stanford University Medical Center, Stanford, USA; 7 Department of Radiology, University of California, San Diego, USA; 8 Department of Radiology, Vanderbilt University, Medical Center North, Nashville, USA; 9 Department of Nuclear Medicine, University Hospital Bonn, Bonn, Germany; 10 Division of Vascular and Interventional Radiology, Icahn School of Medicine at Mount Sinai, New York, USA

**Keywords:** Dosimetry, Radioembolisation, Liver, Guidelines, Yttrium

## Abstract

**Introduction:**

Guidelines on how to adjust activity in patients with a history of liver surgery who are undergoing yttrium-90 radioembolisation (^90^Y-RE) are lacking. The aim was to study the variability in activity prescription in these patients, between centres with extensive experience using resin microspheres ^90^Y-RE, and to draw recommendations on activity prescription based on an expert consensus.

**Methods:**

The variability in activity prescription between centres was investigated by a survey of international experts in the field of ^90^Y-RE. Six representative post-surgical patients (i.e. comparable activity prescription, different outcome) were selected. Information on patients’ disease characteristics and data needed for activity calculation was presented to the expert panel. Reported was the used method for activity prescription and whether, how and why activity reduction was found indicated.

**Results:**

Ten experts took part in the survey. Recommendations on activity reduction were highly variable between the expert panel. The median intra-patient range was 44 Gy (range 18–55 Gy). Reductions in prescribed activity were recommended in 68% of the cases. In consensus, a maximum D_Target_ of 50 Gy was recommended.

**Conclusion:**

With a current lack of guidelines, large variability in activity prescription in post-surgical patients undergoing ^90^Y-RE exists. In consensus, D_Target_ ≤50 Gy is recommended.

***Key points*:**

• *BSA method does not account for a decreased remnant liver volume after surgery.*

• *In post-surgical patients, a volume-based activity determination method is recommended.*

• *In post-surgical patients, a mean D*_*Target*_ *of ≤ 50Gy should be aimed for.*

**Electronic supplementary material:**

The online version of this article (doi:10.1007/s00330-017-4889-6) contains supplementary material, which is available to authorized users.

## Introduction

Liver surgery is often the preferred treatment option for patients with primary or secondary liver malignancies [1]. Unfortunately, primary curative liver resection is only feasible in a small minority of patients [1], and intrahepatic recurrence is commonly seen during follow-up [2]. When not amenable for repeated liver surgery, patients are often directed towards palliative therapies.

Hepatic ^90^Y radioembolisation (RE) with resin microspheres (SIR-Spheres®, Sirtex, Sydney, Australia) is an established treatment modality for both primary and secondary liver malignancies, and is considered to be safe and effective in patients with non-resectable hepatic malignancies [3].

Activity calculation [in Becquerel (Bq)] for ^90^Y-RE is important for accurate treatment, in terms of an effective absorbed dose [in Gray (Gy)] to the tumour, while confirming a safe absorbed dose to the healthy liver tissue [3]. Ideally, activity calculation should be based on accurate and predictive pre-treatment dosimetry [4]. However, when using ^90^Y resin microspheres, international guidelines have historically recommended the simple and semi-empirical body surface area (BSA) method for activity prescription [5–7].

Although the BSA method is popular due to its simplicity and is generally safe within the context of its original design [6], serious limitations for personalised dosimetry have come to light [8, 9]. Studies have shown that BSA does not correlate well to the liver volume or tumour dosimetry, especially in situations of very high and very low tumour burden [9, 10]. Logically, the lack of correlation between BSA and liver volume results in even higher ambiguity in patients with a history of liver surgery because of the reduced remnant liver volume (RLV) [11, 12]. This scenario represents a significant number of patients currently receiving ^90^Y-RE [13–15], potentially leading to overdosing. In order to minimise the risk of clinically significant radioembolisation-induced liver disease (REILD), empirical dose reduction has been recommended for cases of small RLV [16]. Nevertheless, guidelines on how to adjust the prescribed activity in patients with a history of liver surgery are currently lacking.

The primary aim of this study was to study the variability in activity prescription in patients with a history of liver surgery between centres with extensive experience using resin microspheres ^90^Y-RE, and to draw recommendations on activity prescription based on an expert panel survey.

## Materials and methods

For this study, the patients’ data was retrospectively collected. The study was reviewed by the institutional review board, and the requirement to obtain informed consent was waived. Records of patients undergoing ^90^Y-RE with resin microspheres between February 2009 and July 2015 were screened and patients with a history of liver surgery were selected.

## Expert panel

For the survey on current clinical practice, a selection was made of post-surgical patients, based on their clinical history and post-treatment outcome. The selected patients presented a homogenous group in terms of primary malignancy and approximately comparable prescribed activity, but different surgical history and post-treatment outcome in terms of toxicity. The main goal of this selection was to reach sufficient patient variability in terms of remnant liver volumes and treatment outcome, in order to detect whether, how and why activity reduction was applied. All cases were presented to 10 international experts in the field of ^90^Y RE, including 9 high-volume centres. The expert panel members were all internationally recognized experts on ^90^Y RE. Included in the expert panel were six (interventional) radiologists, three nuclear medicine physicians (including the corresponding author) and one hepatologist. Patients’ disease characteristics were summarised and representative imaging (CT and ^99m^Tc-MAA) were provided as well as all necessary data needed for activity calculation. The aim was to provide the expert panel members with all data needed for dosimetry calculation according to the current daily practice. They were blinded for treatment outcome.

## Endpoint

The panel was primarily asked to prescribe activity based on expert opinion (along with an explanation of the rationale for any activity adjustment), and was blinded to the actual activity administration, and also to clinical outcome. The primary endpoint was the variability in prescribed activity for each case.

## Procedure

All procedures were performed by experienced interventional radiologists (with at least 5 years of experience) according to standard recommendations [3]. The imaging workup prior to the ^90^Y-RE procedure required at least contrast-enhanced multiphase CT or MRI, with or without fluorine-18 (^18^F) fluorodeoxyglucose (^18^F-FDG) positron emission tomography/computed tomography (PET/CT). Technetium-99 m macroaggregated albumin (^99m^Tc-MAA) was used for simulation imaging after coil embolisation of relevant hepatico-enteric vessels during preparatory angiography. Activity calculations were performed in compliance with international consensus guidelines and the package insert of resin microspheres [3, 7]. The prescribed activity was calculated in GBq by the following equation = BSA (m^2^) – 0.2 + % tumour involvement/100 [7].

In case of significant hepatopulmonary shunting (>30 Gy lung-absorbed dose), the prescribed activity was empirically reduced using standard reference tables provided by the manufacturer. The net administered activity was determined by correcting the prepared activity for residual activity in the v-vial and tubing. Follow-up of patients consisted of evaluation of the treatment by means of telephone consultation at 2 weeks, clinical and laboratory examination at 4 weeks and imaging follow-up at 3 months. All adverse events were reported and toxicity was graded according to Common Terminology Criteria for Adverse Events (CTCAE) version 4.03 [17].

## Dosimetry

We defined ‘target-absorbed dose’ (D_Target_) as the mean absorbed dose (Gy) across the entire treated arterial territory, regardless of the natural microsphere heterogeneity between tumour and non-tumorous tissue. D_Target_ was retrospectively calculated using the medical internal radiation dose (MIRD) formula [18]. CT imaging was used to calculate the target volume, which was converted to mass (M in kg) using an assumed soft-tissue density of 1.06 g/ml. Homogeneous distribution of the microspheres in the target volume (including tumour and liver parenchyma) was assumed for calculating the D_Target_ utilising the following equation:

$$ {D}_{Target}(Gy)=\left[{A}_{Y90}(GBq)\ /\  M(kg)\right]*50\left( J/ GBq\right), $$

where A_Y90_ is the administered activity corrected for lung shunt.

## Statistical analysis

Descriptive analyses were performed to summarize patient demographics and treatment characteristics. The variability in prescribed activity in each case was illustrated graphically. The variability was quantified by reporting the range of prescribed activity for each case and comparing it with the BSA-based prescribed activity. A commercial statistical software package (SPSS for Windows, version 19.0; SPSS Inc, Chicago, IL, USA) was used for data analysis.

## Results

### Patient population

Between February 2009 and July 2015, 187 consecutive patients with primary and secondary liver malignancies underwent ^90^Y-RE with resin microspheres in our clinic. Forty-three patients had a history of liver surgery varying from ablative therapy to an extended hemihepatectomy. Six patients were found eligible for the expert panel survey (Table 1). Five out of the six patients received ^90^Y-RE for colorectal liver metastases and one patient received treatment for cholangiocarcinoma. In all patients, the entire remnant liver was treated. The data used for activity calculation and presented to the expert panel is presented in Table 2.

**Table 1:** Patient characteristics selected for the expert panel

Case	Age	Sex	WHO	Child–Pugh score	Primary tumour	Previous systemic therapy	Previous surgery	Laboratory findings
1	60	M	2	A5	CRC	1st line chemotherapy (1× CAPOX)	Right-sided hemihepatectomy 2x segment resection IVa	Bili 0.82 mg/dL, ALP 54 U/L, GGT 29 U/L, ASAT 32 U/L, ALAT 25 U/L, alb 40.4 g/L
2	57	F	1	A5	CRC	1st line chemotherapy (2× CAPOX) 2nd line chemotherapy (5× CAPOX)	2 operations: liver resection segment VII, liver resection segment III + VIII	Bili 0.35 mg/dL, ALP 102 U/L, GGT 98 U/L, ASAT 38 U/L, ALAT 24 U/L, alb 40.7 g/L
3	72	M	0	A5	CRC	1st chemotherapy (CAPOX, FOLFOX)	Portal vein embolisation, Extended right-sided hemihepatectomy	Bili 0.64 mg/dL, ALP 159 U/L, GGT 342 U/L, ASAT 59 U/L, ALAT 52 U/L, alb 42.5 g/L
4	74	F	1	A5	CRC	1st line chemotherapy (3× CAPOX) 2nd line chemotherapy (capecitabine + bevacizumab)	Extended right-sided hemihepatectomy	Bili 0.82 mg/dL, ALP 141 U/L, GGT 159 U/L, ASAT 29 U/L, ALAT 26 U/L, alb 39.9 g/L, thrombocytes 126 x10^9^/L
5	46	M	0	A5	CRC	1st line + 2nd line chemotherapy (9× CAPOX + bevacizumab)	RFA on 13 lesions	Bili 0.35 mg/dL, ALP 568 U/L, GGT 584 U/L ASAT 50 U/L ALAT 126 U/L, alb 40.9 g/L
6	63	M	0	A5	CC	1st line chemotherapy (gemcitabine + cisplatin) 2nd line chemotherapy (3× gemcitabine + cisplatin)	Right-sided hemihepatectomy	Bili 0.82 mg/dL, ALP 118 U/L, GGT 353 U/L ASAT 45 U/L, alb 41.2 g/L, Hb 8.1 mmol/L, thrombocytes 94x10^9^/L

*ALAT* alanine transaminase, *Alb* Albumin, *ALP* alkaline phosphatase, *ASAT* aspartate aminotransferase, *Bili* bilirubin, *CAPOX* capecitabine + oxaliplatin, *CRC* colorectal carcinoma, *CC* cholangiocarcinoma, *FOLFOX* folinic acid, fluorouracil, oxaliplatin, *GGT* gamma-glutamyl transferase, *RFA* radiofrequency ablation.

**Table 2:** Data of patients selected for the expert panel that was used for activity calculation based on the BSA method

Case	BSA (m^2^)	Target volume (mL)	tumour burden (%)	Lung shunt (%)	Activity based on BSA (GBq)	Target dose based on BSA (Gy)	Target dose in case of reduction (Gy)^#^	Net delivered dose (Gy)*
1	2.06	1654	3	8	1.89	54	-	47
2	2.17	2306	10	1	2.07	42	-	40
3	1.91	1091	5	3	1.76	76	50	44
4	1.79	1218	15	5	1.74	70	50	43
5	2.05	1894	6	4	1.91	48	-	45
6	1.90	1091	6	4	1.76	76	57	54

^#^The net target absorbed dose that was delivered after reduction: 50 Gy target absorbed dose in case 3 and 4, 25% reduction in case 6.

^*^The net delivered target absorbed dose is the mean absorbed dose (Gy) across the entire treated arterial territory calculated by the net administered activity.

For comparison between cases and experts, all prescribed activities were reported as target-absorbed dose or D_Target_, using prescribed activities and target volumes as described above. The actual prescribed activity ranged from 1.74 to 2.07 GBq and resulted in a D_Target_ of 42–76 Gy. In three out of six patients (cases 3, 4 and 6), the delivered activity was reduced due to previous liver resection. The lowest net delivered D_Target_ was 40 Gy in case 2 with RLV of 2306 mL, and the largest net delivered D_Target_ was 54 Gy in case 6, with a RLV of 1091 mL (Table 2).

### Expert review and correlation

**Fig. 1:**
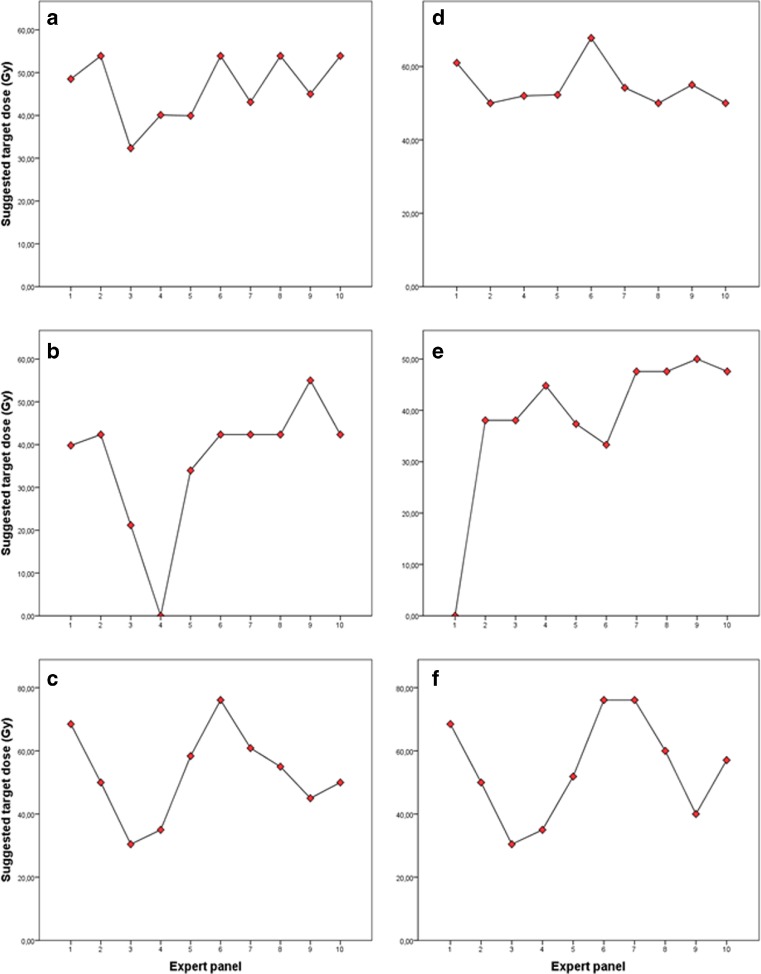
The target absorbed dose (Gy) as recommended by the expert panel for each case (A–F). The median (and range) recommended absorbed dose for each case was 49 Gy, range 32–54 (A), 42 Gy, range 0–55 Gy (B), 55 Gy, range 30–76 Gy (C), 53 Gy, range 50–68 Gy (D), 46 Gy, range 0–50 Gy (E) and 59 Gy, range 35–76 Gy (F). Note: 0 Gy means that the expert advised not to treat that specific patient at all.

The recommended activity adjustment for individual patients was highly variable and there was no consensus for any of the cases (Figure 1). The median intra-patient range (i.e. the highest minus the lowest recommended D_Target_) was 44 Gy (range 18–55 Gy). Reductions were reported 41 out of 60 times (68%), no treatment at all in 2 out of 60 times (3%), and there was one missing in the survey (2%; Table 3). Different methods were used to calculate the prescribed activity in cases of reduction. For most reductions (17/41; 41%), the experts chose to empirically reduce the activity as a percentage of the prescribed activity based on the BSA method (median 20%, range 10–60%). In other cases (15/41; 37%) a pre-set maximum safe D_Target_ was recommended (median 50 Gy, range 35–55 Gy).The partition model was recommended 3 out of 41 times (7%). In these cases, the recommended D_tumour_, D_Lung_ and maximum dose on healthy liver were given (median 40 Gy, range 40–54 Gy). Finally, in 6 out of 41 reductions (15%), an empirically prescribed activity was recommended (median 46 Gy, range 34–58 Gy; Table 3). Repeatedly, >1 reason for dose reduction was mentioned, resulting in a total of 65 reported reasons. Small RLV was the most frequent reason for activity reduction (24/65; 37%). Other arguments for reduction were: previous systemic therapy (16/65; 24%), low tumour burden (10/65; 15%), findings of unfavourable microsphere biodistribution on ^99m^Tc-MAA SPECT (8/65; 12%), altered liver function (5/65; 8%), previous radiotherapy (1/65; 2%), and performance status (1/65; 2%).

**Table 3:** The prescribed D_Target_ (Gy) for each case as advised by the expert panel

		Case 1	Case 2	Case 3	Case 4	Case 5	Case 6
Expert 1	Type D_Target_ (Gy)	PC 49	PC 39	PC 68	PC 63	No treatment	PC 68
Expert 2	Type D_Target_ (Gy)	NR 54	NR 42	MIRD 50	MIRD 50	PC 38	MIRD 50
Expert 3	Type D_Target_ (Gy)	PC 32	PC 21	PC 30	*Missing*	PC 38	PC 30
Expert 4	Type D_Target_ (Gy)	PM 40	No Treatment	MIRD 35	PM 54	PM 40	MIRD 35
Expert 5	Type D_Target_ (Gy)	EPA 40	EPA 34	EPA 58	EPA 54	EPA 37	EPA 52
Expert 6	Type D_Target_ (Gy)	NR 54	NR 42	NR 76	NR 70	PC 34	NR 76
Expert 7	Type D_Target_ (Gy)	PC 43	NR 42	PC 61	PC 56	NR 48	NR 76
Expert 8	Type D_Target_ (Gy)	NR 54	NR 42	MIRD 50-60	MIRD 50-60	NR 48	PC 60
Expert 9	Type D_Target_ (Gy)	MIRD 45	MIRD 55	MIRD 45	MIRD 55	MIRD 50	MIRD 40
Expert 10	Type D_Target_ (Gy)	NR 54	NR 42	MIRD 50	MIRD 50	NR 48	PC 57

*EPA* Empirically prescribed activity, *MIRD* Maximum safe whole liver absorbed dose (D_Target_) based on Medical Internal Radiation Dose (MIRD) dosimetry, *NR* no reduction was recommended, *PC* Empirical reduction of the activity as a percentage of the prescribed activity based on the BSA method, *PM* Partition model with prescribed D_Tumour_, D_HL_ and D_Lung_ (the tumour to non-tumour ratio was assumed).

### Toxicity and outcome

**Fig. 2:**
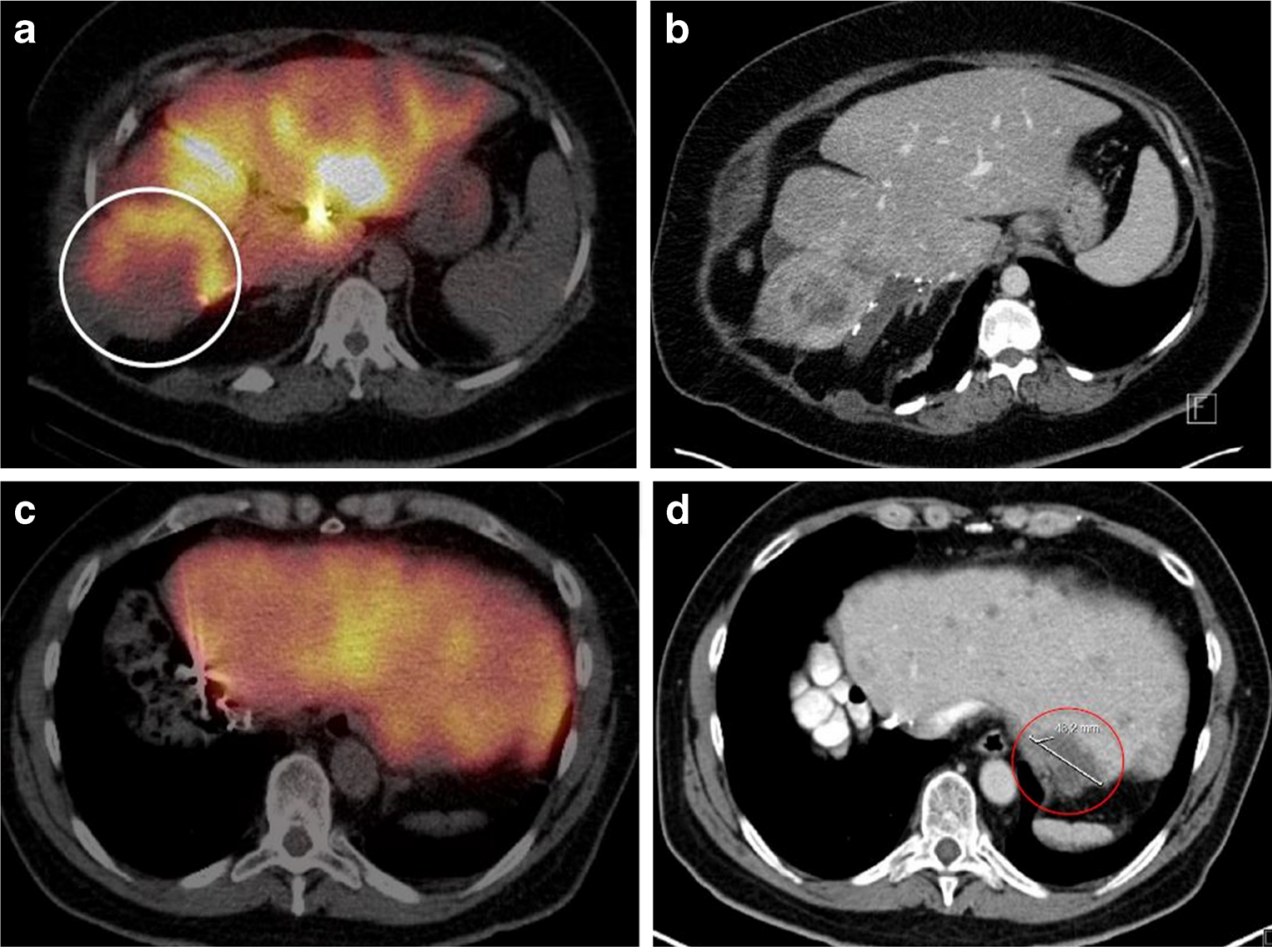
The ^99m^Tc-MAA SPECT (A and C) and the pre-procedural CT imaging (B and D) of, respectively, cases 2 and 6 as presented to the expert panel along with following information: the ^99m^Tc-MAA SPECT of case 2 shows low activity around the largest lesion in segment VIII and relatively high uptake in the healthy liver parenchyma after injection of MAA in the left hepatic artery (A and B). In case 6, the ^99m^Tc-MAA SPECT and the pre-procedural CT imaging show distribution of the activity throughout the remnant liver, although with lack of activity uptake around the largest lesion as depicted by the *red circle* (C and D).

Post-treatment clinical symptoms were generally minor and included transient nausea, pain and fatigue related to the post-embolisation syndrome (Table 4). REILD was seen in one patient (case 6) who developed grade 3 ascites with elevated liver biochemistry tests (with grade 1 hyperbilirubinemia at last measurement) and died 71 days after the treatment. The remaining cases developed only mild clinical and laboratory symptoms. With regards to disease outcome, case 2 was the first to develop progressive disease (within 3 months) and died 10 days after treatment due to progressive disease. Notably, the patient who developed REILD (case 6) had the smallest RLV and received the highest net D_Target_, while the latter patient (case 2) had the largest RLV and received the smallest D_Target_. The ^99m^Tc-MAA SPECT images and the pre-procedural CT imaging of these two cases are shown in Figure 2.

**Table 4:** Reported adverse events within 3 months following the ^90^Y RE procedure according to the CTCAE version 4.03 for the six cases presented to the expert panel

Adverse events	Laboratory toxicity*	Clinical toxicity
Case 1	ALP (+1 CTC) GGT (+3 CTC) ASAT (+1 CTC) ALAT (+1 CTC)	Fatigue (CTC 1) Nausea (CTC 1)
Case 2	ALP (+1 CTC) GGT (+1 CTC)	Fatigue (CTC 2) Nausea (CTC 1) Pain (CTC 2)
Case 3	Bili (+2 CTC) ALP (+1 CTC) ASAT (+1 CTC)	Fatigue (CTC 1) Nausea (CTC 1) Pain (CTC 1)
Case 4	GGT (+1 CTC) ASAT (+1 CTC) ALAT (+1 CTC)	None
Case 5	Bili (+1 CTC)	Fatigue (CTC 1) Pain (CTC 1) Jaundice
Case 6	Bili (+1 CTC) ALP (+1 CTC) ALAT (+1 CTC) Albumin (+2 CTC)	Pain (CTC 2) Ascites (CTC 2)

*ALAT* alanine transaminase, *ALP* alkaline phosphatase, *ASAT* aspartate aminotransferase; Bili: bilirubin, *CTC* common toxicity criteria, *GGT* gamma-glutamyl transferase.

*Laboratory toxicity is reported as highest increase in CTC toxicity grade compared to baseline CTC grade (+1 etc.)

## Discussion

In general, ^90^Y-RE resin microspheres are safe for most patients with a history of liver surgery. However, the currently advocated BSA method for activity calculation does not incorporate the actual liver volume. In patients with extensive liver surgery, the remnant liver volume may have been significantly reduced, leading to potential overdosing in case this is not accounted for. Remarkably, there have been no publications on the effects of dosimetry on the outcome in terms of treatment response and toxicity in post-surgical patients. Since guidelines are lacking, the variability in used methods to reduce activity may be large. Consequently, administration of excessive activities to the normal liver parenchyma in post-surgical patients does occur, leading to fatal REILD in some cases. This study demonstrated that in current clinical practice, there is no consensus regarding the preferred method of adjustment of the prescribed activity in patients with a history of liver surgery. These findings raise some safety concerns for ^90^Y-RE after liver surgery.

Only a few studies have investigated the safety of ^90^Y-RE resin microspheres in patients with a history of liver surgery [13–15]. In all of these studies, activity calculation was based on the BSA method. It was, therefore, suggested that this method can safely be used in post-surgical patients. However, controversy remains, as it has been shown that the BSA model does not correlate well with liver volume, specifically in large and/or obese patients [8, 9]. Furthermore, patients that have undergone hepatic surgery pose an even greater challenge due to altered liver volume secondary to regeneration [9, 10], and due to a reduced liver functional reserve that may result in an increased risk of REILD. Significantly higher activity concentrations in the normal liver parenchyma can be measured in patients with pre-existent smaller liver volumes, or reduced liver volumes [10]. The cases of the expert panel survey showed significant toxicity in case of a high D_Target_ (Gy) in a patient with a relatively small RLV (i.e. overdosing), and suggested underdosing in case of a small D_Target_ (Gy) in patients with a relatively large RLV (and comparable BSA). This finding again illustrates the lack of correlation between BSA and the (remnant) liver volume.

The results of the survey illustrated the lack of consensus regarding the preferred method of activity reduction in any of the cases. Moreover, different methods were used for activity adjustment. In a high percentage of cases, the expert panel agreed on the fact that a low RLV is an argument for careful use of the BSA method, due to the high risk of an overdose. Preferably, a specific D_Target_ was prescribed (frequently ±50 Gy), and in some cases, the D_Target_ was further specified in terms of absorbed dose to the tumour tissue, non-tumour tissue and the lungs, according to the ‘partition model’ which is based on MIRD macrodosimetry [6, 18, 19]. Clinical explanations may be provided for the differences in activity prescription provided by the expert panel. The patients who are eligible for ^90^Y-RE treatment represent a heterogeneous group with distinct disease characteristics. There is significant diversity in the patients’ medical histories, such as prior chemotherapy, which is known to increase the risk of REILD [20]. Also, tumour vascularity may differ, which influences the microsphere biodistribution of the delivered activity to tumour and non-tumour tissue [8]. It has been mentioned previously that the BSA method neglects the inter-patient variability in tumour-to-normal liver tissue microsphere biodistribution [6, 19], a possible argument for activity adjustment. Lastly, the extent of liver surgery differs highly, resulting in large differences between remnant liver volumes.

A limitation of this study is the retrospective selection of patients in the survey. However, the selected patients were representative for daily practice, with risk of under- and overdosing in patients with similar prescribed activity (based on the BSA method) but distinct RLV and DTarget. Hence, the lack of guidelines for dosimetry, especially in this patient group, directly affects clinical outcome and should be investigated more thoroughly.

Based on these results, the blind application of the BSA method for activity prescription in patients with previous liver surgery is discouraged. Based on the reports of the expert panel, the following consensus recommendation can be made; for activity calculation of ^90^Y-RE using resin microspheres, a volume-based activity calculation algorithm is recommended, such as the MIRD method that takes RLV into account. A maximum D_Target_ of 50 Gy seems a safe interim recommendation, with further reduction in the case of relatively hypovascular tumours, dismal baseline liver function, or a heavily pre-treated patient. The partition model may also warrant further consideration and research as it theoretically takes into account the differential activity biodistribution to both the tumour and the normal liver tissue [19, 21]. As regards to a recommended safe cut-off value for the dose on healthy liver tissue, different possible safe cut-off points have been reported [6, 22]. However, in view of the applicability of the partition model for individualised dosimetry, more research on this topic is essential. Finally, in order to facilitate patient-specific dosimetry of ^90^Y-RE treatment and future research on this topic, accessible tools have been developed [23].

## Conclusion

Caution should be taken when using the BSA method for activity prescription in patients with previous liver resection. Based on the survey of the expert panel, an interim recommendation is to use a volume-based activity determination method such as the MIRD-based model instead, aiming for a mean absorbed dose across the target arterial territory (D_Target_) of ≤50 Gy, until the emergence of more robust dosimetric data. Partition modelling should lead to further refinement in personalised activity calculation based on healthy liver and lung-absorbed dose thresholds.

## Electronic supplementary material

Below is the link to the electronic supplementary material.

ESM 1 (PDF 1364 kb)
