# Supplementary material for: Recommendations for radioembolisation after liver surgery using yttrium-90 resin microspheres based on a survey of an international expert panel
Source: Eur Radiol. 2017 Jul 3;27(12):4923–30. doi: 10.1007/s00330-017-4889-6 (PMC5674129; doi:10.1007/s00330-017-4889-6)
Supplement: Supplementary file 1 — (PDF 1364 kb) [file 330_2017_4889_MOESM1_ESM.pdf]

Case 1: hemi-hepatectomy right

- Male, 60 yr.
- Oncology:
  - 2009 Colorectal carcinoma TNM classification unknown, 1st line chemotherapy (1x CAPOX)
  - 2009 Hemi-colectomy right, followed by hemi-hepatectomy right + 2 metastasectomies segment 4a
- Laboratory: bili 0.82 mg/dL, ALP 54 U/L, GGT 29 U/L, AST 32 U/L, ALT 25 U/L, alb 40.4 g/L. No other relevant findings
- WHO grade 2, Child Pugh A5
- 8 February 2011 CT:
  - Classical vascular anatomy
- 22 February 2011 MAA:
  - BSA 2.06, lung shunt 8%
  - Total liver volume 1654 ml, tumour burden 3%

- A_{Y90} *(GBq) = [(BSA - 0.2) +* TI_{liver}*] = (2.06-0.2)+0.03 = 1.89 GBq*
- *The aim is to treat the whole remnant liver volume in one session*

Imaging

History of right hemi-hepatectomy. The ^{99m}TC-MAA SPECT images and the related pre-procedural CT images showing relatively low activity around the lesions in segments 2 and 3 and preferential uptake in segment 4 after injection of MAA in the left hepatic artery.

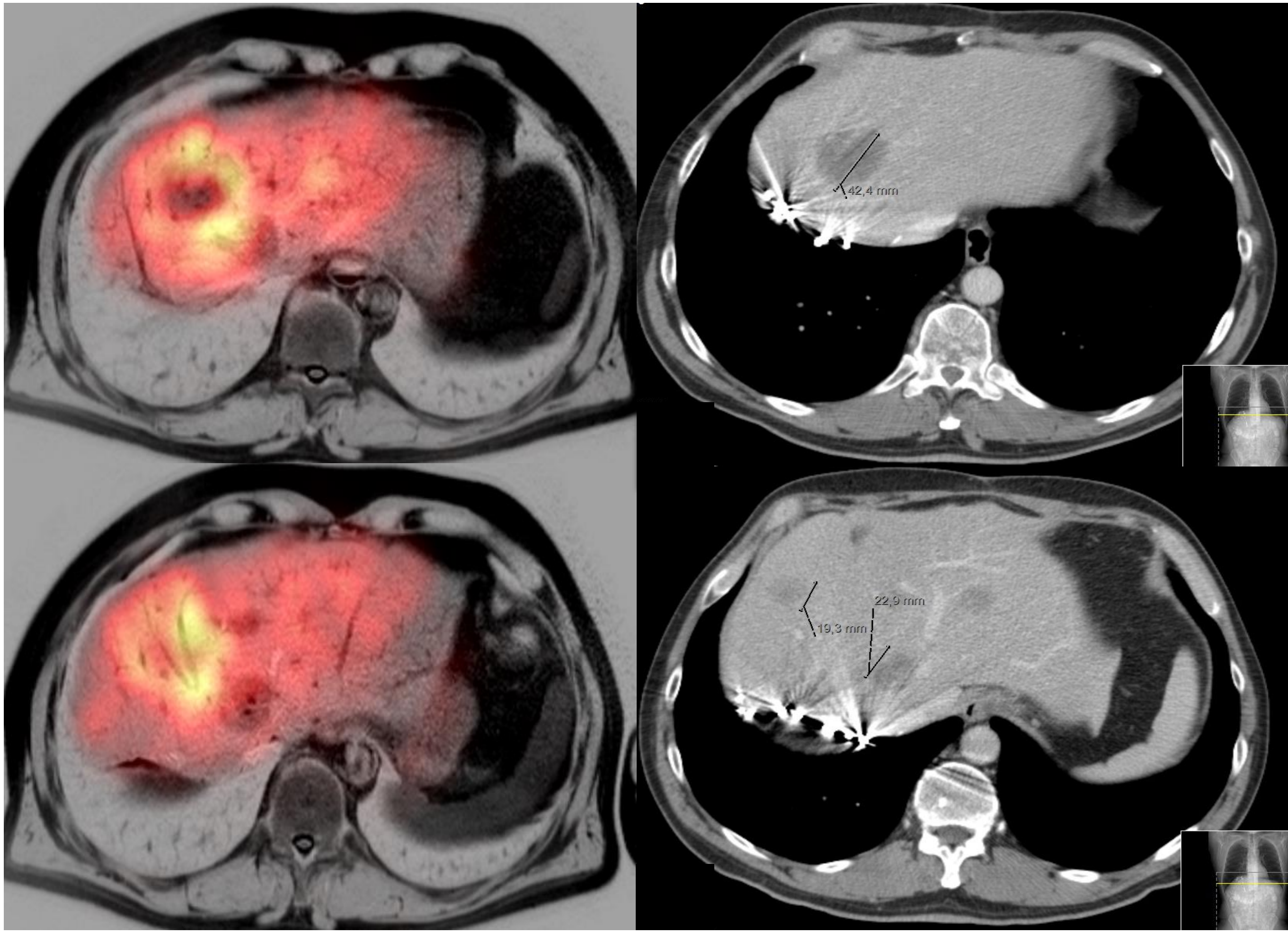

Pre-procedural CT images showing multiple lesions in the left liver lobe. Largest lesion measures 42 mm (top). The bottom pictures shows two lesions of respectively 23 and 13 mm.

Case 2: resection of individual liver metastasis

- Female, 57 yr.
- Oncology:
  - 2008 Colorectal carcinoma pT3N1M1: sigmoid resection + 1st line chemotherapy (3x CAPOX)
  - 2009 2x resection liver metastasis segment 7, 2011 resection liver metastasis segment 3 + 8
  - 2012 progression liver metastasis: 2nd line chemotherapy (5x CAPOX)
- Laboratory: bili 0.35 mg/dL, ALP 102 U/L, GGT 98 U/L, AST 38 U/L, ALT 24 U/L, alb 40.7 g/L. No other relevant findings
- WHO grade 1, Child Pugh A5
- 29 January 2013 CT:
  - Abnormal vascular anatomy: early branching RHA from CHA
- 26 February 2013 MAA:
  - BSA 2.17, lung shunt fraction 1%
  - Total liver volume 2306 ml, tumour burden 10%

- A_{Y90} *(GBq) = [(BSA - 0.2) +* TI_{liver}*] = (2.17-0.2)+0.1 = 2.07 GBq*
- *The aim is to treat the whole remnant liver volume in one session*

Imaging

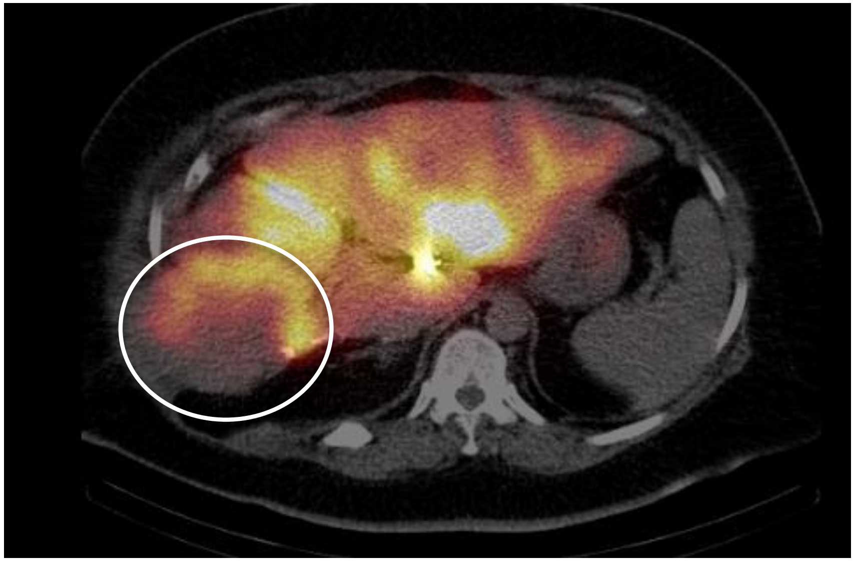

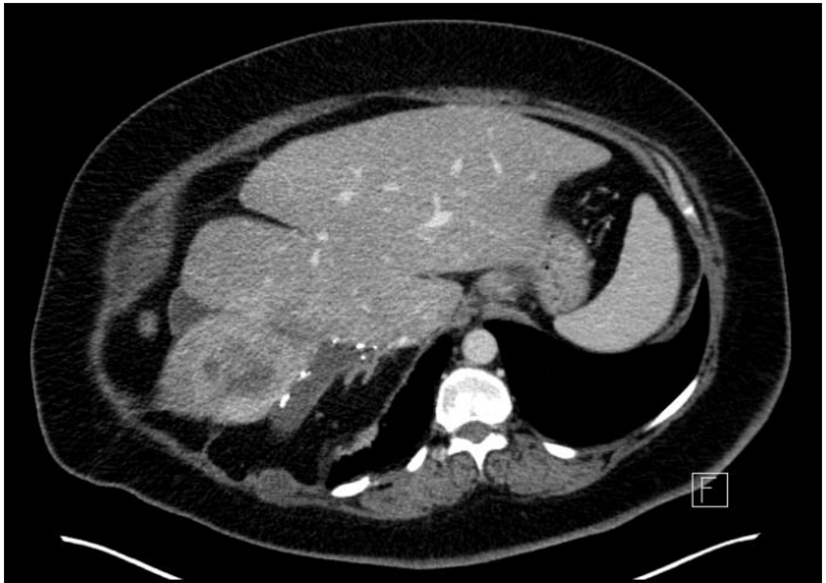

History of liver segment resection. The ^{99m}Tc-MAA SPECT image and the related pre-procedural CT image (top right) showing low activity around the largest lesion in segment 8 (white circle) and relatively high uptake in the healthy liver parenchyma after injection of MAA in the left and right hepatic artery.

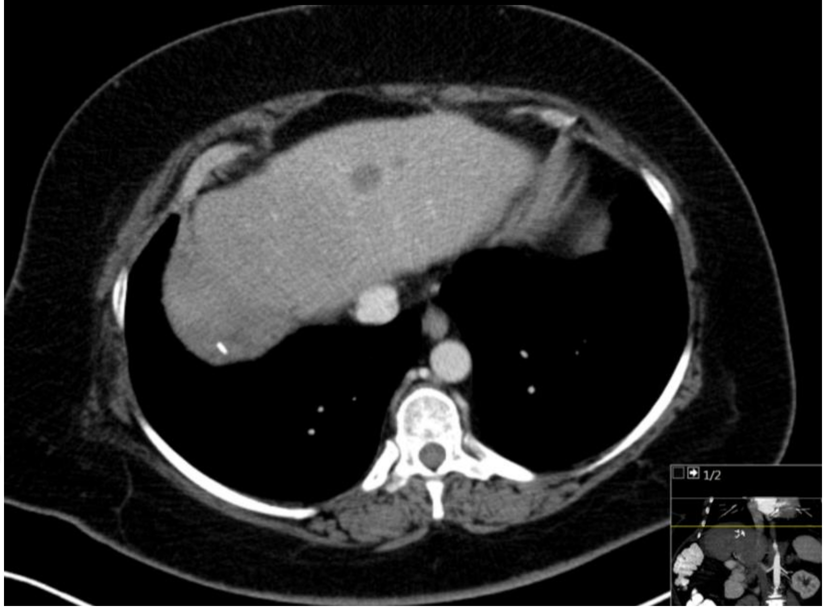

Pre-procedural CT images. The largest lesion is located in segment 8 (top) and two small hypodense lesions in segments 2 and 3 (bottom).

Case 3: extended hemi-hepatectomy right

- Male, 72 yr.
- Oncology:
  - 2008 Colorectal carcinoma pT4N2M0: hemi-colectomy right + 1st chemotherapy (CAPOX, FOLFOX, due to toxicity switched to 5FU-Leucovorin)
  - 2012 v. porta embolization right, extended hemi-hepatectomy right (remnant liver segment 1-3)
- Laboratory: bili 0.64 mg/dL, ALP 159 U/L, GGT 342 U/L, AST 59 U/L, ALT 52 U/L, alb 42.5 g/L. No other relevant findings
- WHO grade 0, Child Pugh A5
- 31 March 2014 CT:
  - Aberrant vascular anatomy: replaced LHA S2-3
- 15 April 2014 MAA:
  - BSA 1.91, lung shunt fraction 3%
  - Total liver volume 1091 ml, tumour burden 5%

- $A_{Y90}\ (GBq) = [(BSA - 0.2) + TI_{liver}] = (1.91-0.2)+0.05 = 1.76\ GBq$
- *The aim is to treat the whole remnant liver volume in one session*

Imaging

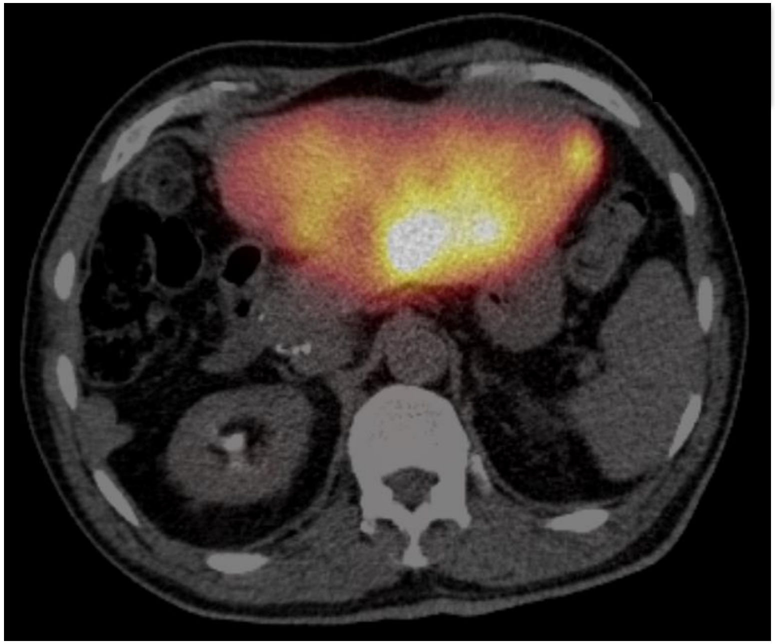

History of right hemi-hepatectomy. The ^{99m}Tc-MAA SPECT image and the related pre-procedural CT image showing preferential activity around the lesion in segment 3 and relatively low activity in segment 1 after injection of MAA in the replaced left hepatic artery.

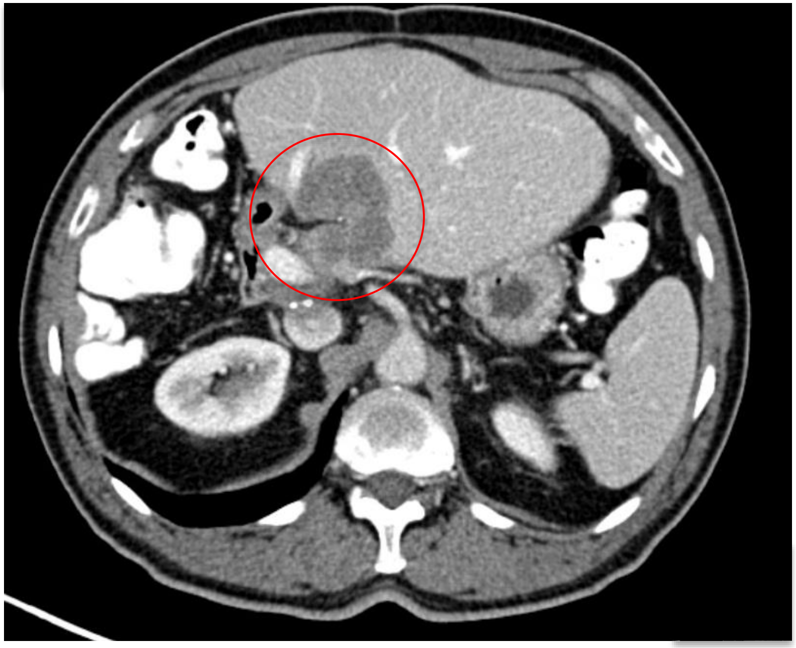

Pre-procedural CT images showing the remnant liver (segments 2,3 and 1). Largest lesion is located in segment 3 and 1 and measures 45 mm (red circle).

Case 4: extended hemi-hepatectomy right

- Female, 74 yr.
- Oncology:
  - 2012 Colorectal carcinoma cT3-4N2M1: 1st line chemotherapy (3x CAPOX) and radiotherapy
  - 2013 extended hemi-hepatectomy right and 2nd line chemotherapy (capecitabine + bevacizumab)
- Laboratory: bili 0.82 mg/dL, ALP 141 U/L, GGT 159 U/L, AST 29 U/L, ALT 26 U/L, alb 39.9 g/L, thrombocytes 126 x10^9/L. No other relevant finding
- WHO grade 1, Child Pugh A5
- 9 May 2014 CT:
  - Classical vascular anatomy
- 20 May 2014 MAA:
  - BSA 1.79, lung shunt fraction 5%
  - Total liver volume 1218 ml, tumour burden 15%

- A_{Y90} *(GBq) = [(BSA - 0.2) + TI*$_{liver}$*] = (1.79-0.2)+0.15 = 1.75 GBq*
- *The aim is to treat the whole remnant liver volume in one session*

Imaging

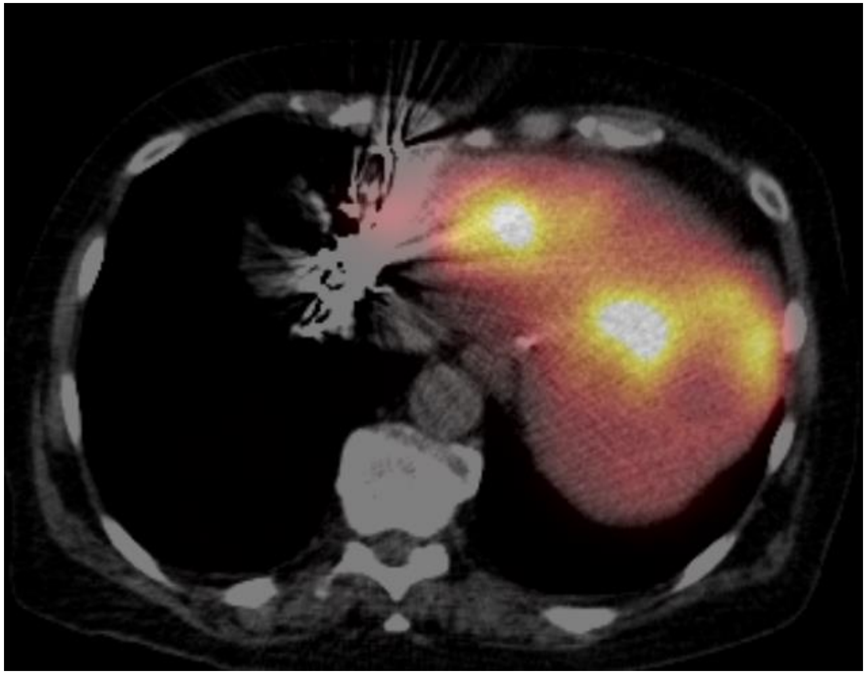

History of extended right hemi-hepatectomy. The ^{99m}Tc-MAA SPECT image and the related pre-procedural CT image showing preferential accumulation of activity around the two tumour lesions in segment 2 after injection of MAA in the replaced left hepatic artery. Note that due to rotation of the liver position during the ^{99m}Tc-MAA SPECT , the hot spots do not match the location of the liver lesions as shown on the CT images.

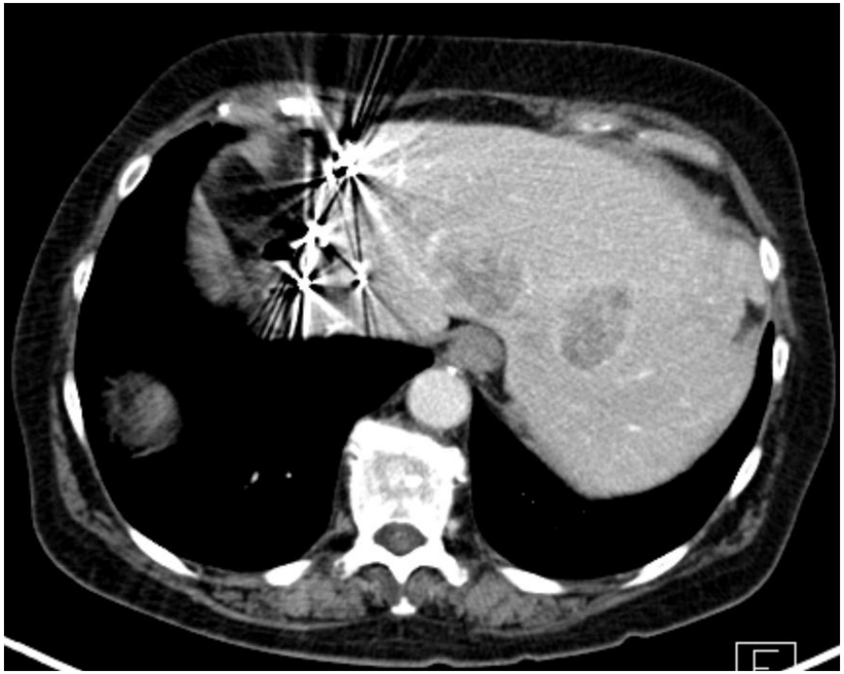

Pre-procedural CT images showing the two tumour lesions in segment 2. Medial lesion is 49 mm and the lateral lesion 37 mm in the long axis.

Case 5: RFA

- Male, 46 yr.
- Oncology:
  - 2010 Colorectal carcinoma: 1st line chemotherapy, RFA liver metastasis (13 locations bilobar) and sigmoid resection followed by 2nd line chemotherapy (9x oxaliplatin+capecitabine+avastin)
- Laboratory: bili total 1.58 mg/dL, direct 0.35 mg/dL, ALP 568 U/L, GGT 584 U/L AST 50 U/L ALT 126 U/L, alb 40.9 g/L. No other relevant findings
- WHO grade 0, Child Pugh A5
- 5 March 2012 CT:
  - Abnormal vascular anatomy: early branching RHA from abdominal aorta
- 27 March 2012 MAA:
  - BSA 2.05 lung shunt fraction 4%
  - Total liver volume 1894 ml, tumour burden 6%

- A_{Y90} *(GBq) = [(BSA - 0.2) + TI_{liver}] = (2.05-0.2)+0.06 = 1.91 GBq*
- *The aim is to treat the whole remnant liver volume in one session*

Imaging

The ^{99m}Tc-MAA SPECT images and the related pre-procedural CT image showing irregular distribution of the activity throughout the liver after injection of MAA in the left and right hepatic artery.

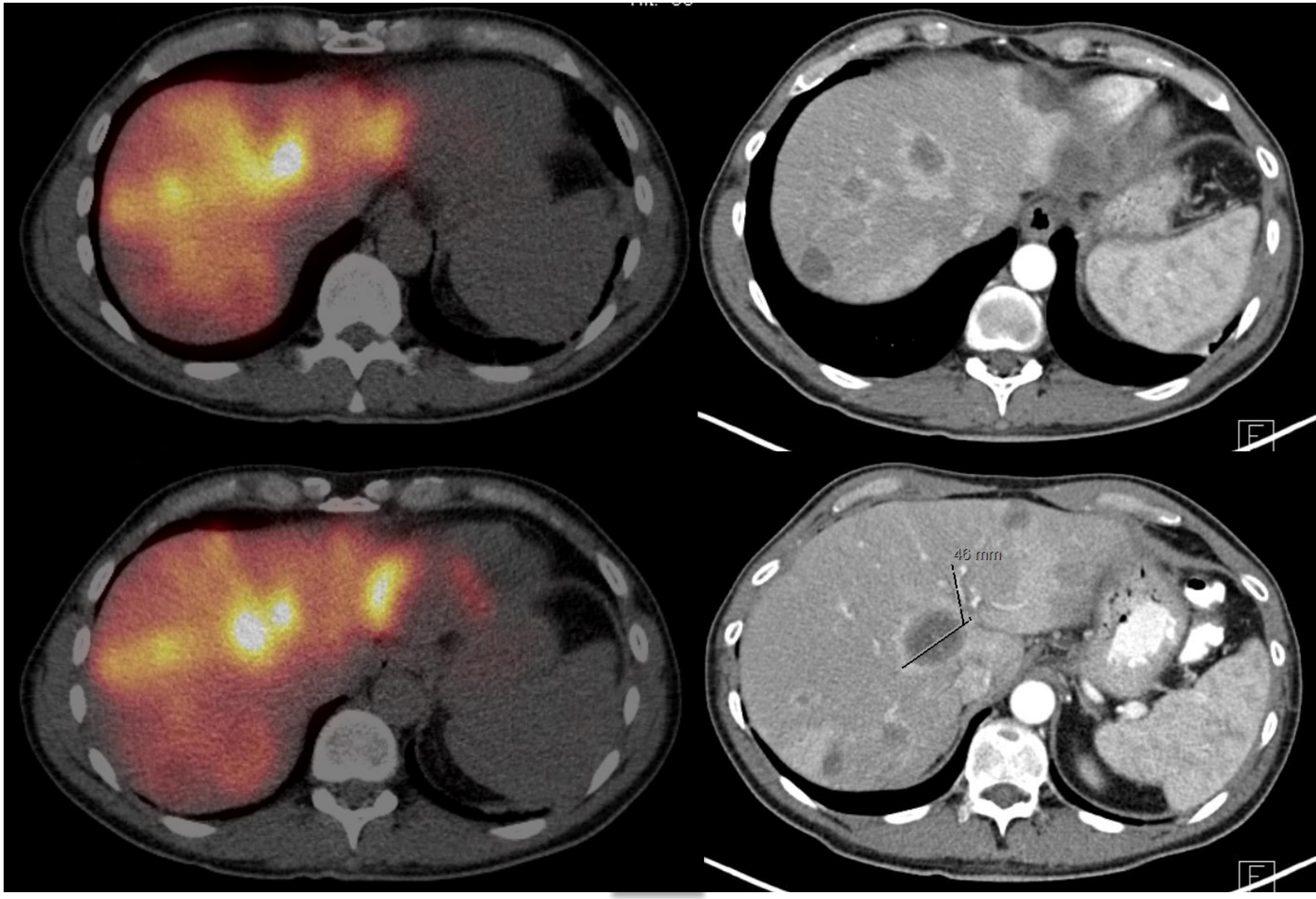

Pre-procedural CT images showing multiple (±12) lesions located in the left and right liver lobe. The largest lesion is 46 mm and located centrally (bottom).

Case 6: hemi-hepatectomy right

- Male, 63 yr.
- Oncology:
  - 2012: cholangiocarcinoma pT3N1M1 cholangectomy+ hemi-hepatectomy right and 1st line chemotherapy (gemcitabine+cisplatin)
  - 2014: 2nd line chemotherapy (3x gemcitabine+cisplatin)
- Laboratory: bili 0.82 mg/dL, ALP 118 U/L, GGT 353 U/L AST 45 U/L, alb 41.2 g/L, Hb 8.1 mmol/L, thrombocytes 94x10^9/L
- WHO grade 0, Child Pugh A5
- 1 August 2014 CT:
  - Classical vascular anatomy
- 19 August 2014 MAA:
  - BSA 1.90, lung shunt fraction 4%
  - Total liver volume 1091 ml, tumour burden 6%

- A_{Y90} *(GBq) = [(BSA - 0.2) +* TI_{liver}*] = (1.9-0.2)+ 0.06 = 1.76 GBq*
- *The aim is to treat the whole remnant liver volume in one session*

Imaging

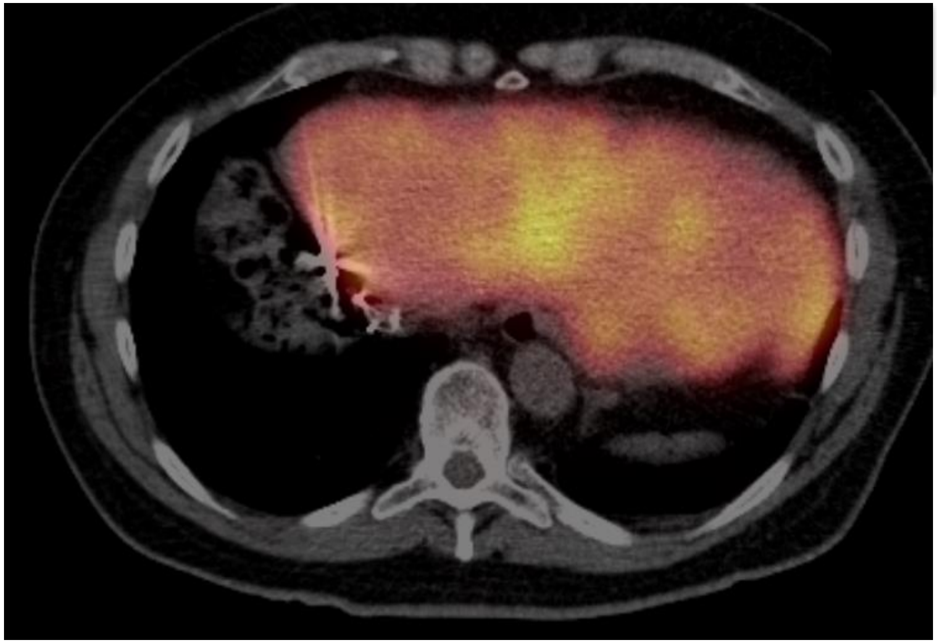

History of extended right hemi-hepatectomy. The ^{99m}Tc-MAA SPECT image and the related pre-procedural CT image showing distribution of the activity throughout the left liver lobe after injection of MAA in the left hepatic artery. Note that there is lack of activity uptake around the largest lesion (red circle on CT image).

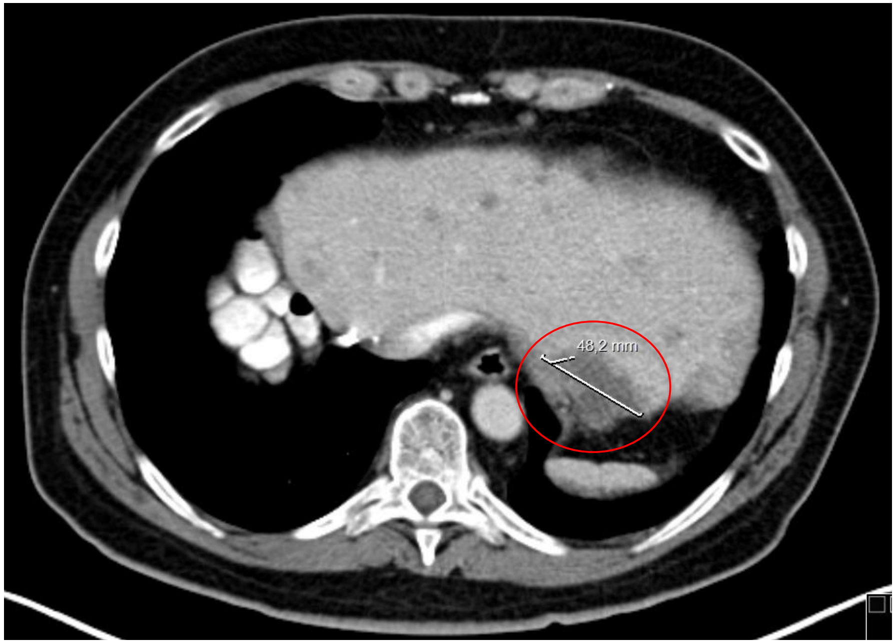

Pre-procedural CT images showing multiple small lesions distributed in the left liver lobe and a large lesion of 48,2 mm adjacent to the resection plane (red circle).

Abbreviations:

Alb: Albumin

ALP: Alkaline phosphatase

ALT: Alanine transaminase

AST: Aspartate transaminase

Bili: Bilirubin

CAPOX: Capecitabine combined with oxaliplatin

FOLFOX: Leucovorin combined with 5-FU and oxaliplatin

FU: Fluorouracil

GGT: Gamma-glutamyl transferase

Hb: Haemoglobin

UMC Utrecht
